# *Toona sinensis* leaf extract has antinociceptive effect comparable with non-steroidal anti-inflammatory agents in mouse writhing test

**DOI:** 10.1186/s12906-015-0599-2

**Published:** 2015-03-19

**Authors:** Yu-feng Su, Yu-Chiao Yang, Hseng-Kuang Hsu, Shiuh-Lin Hwang, Kung-Shing Lee, Ann-Shung Lieu, Te-Fu Chan, Chih-Lung Lin

**Affiliations:** Department of Neurosurgery, Kaohsiung Medical University Hospital, No. 100, Tz-you Road, Kaohsiung, 80708 Taiwan; Department of Pharmacology, Kaohsiung Medical University, Kaohsiung, Taiwan; Department of Physiology, Kaohsiung Medical University, Kaohsiung, Taiwan; Faculty of Medicine, College of Medicine, Kaohsiung Medical University, Kaohsiung, Taiwan; Institute of Clinical Medicine, College of Medicine, Kaohsiung Medical University, Kaohsiung, Taiwan; Department of Obstetrics and Gynecology, Kaohsiung Medical University Hospital, Kaohsiung, Taiwan

**Keywords:** *Toona sinensis*, Visceral pain, Mouse writhing test, Non-steroidal anti-inflammatory drug (NSAID)

## Abstract

**Background:**

The antinociceptive effect of an aqueous extract from the leaves of *Toona sinensis* (TS, [A. Juss., M. Roem.]) was studied using the writhing test in mice.

**Methods:**

Different extraction fractions from TS leaf extracts (TSL1 to TSL5) were administered orally 1 h before intraperitoneal injection of acetic acid.

**Results:**

After treatment with TSL1, TSL2, TSL3, TSL4, and TSL5 at a dose of 1 g/kg, the respective writhing responses were 39.9% (*P <* 0*.*001), 19.9% (*P <* 0*.*05), 11.7% (*P* = 0.052), 8.1% (*P* = 0.188), and 11.4% (*P* = 0.057) lower than the control group. Mice treated with TSL1 at 1 g/kg (39.9%, *P <* 0*.*001), 0.3 g/kg (38.0%, *P <* 0*.*001), 0.1 g/kg (46.9%, *P <* 0*.*001), and 0.03 g/kg (31.1%, *P <* 0*.*001) had significantly lower writhing responses compared with control mice. A time-course experiment was performed, which involved oral administration of TSL1 (0.1 g/kg) at 0, 0.5, 1, 2, and 6 h before acetic acid intraperitoneal injection. The most effective dose of TSL1 was 0.1 g/kg orally, with the effect beginning 30 min before treatment and persisting until 6 h.

**Conclusions:**

This study showed that TS has anti-visceral pain properties comparable with those of rofecoxib (a cyclooxygenase-2 inhibitor) and diclofenac, which suggests promise for the treatment of intractable visceral pain in humans.

## Background

*Toona sinensis* (TS, [A. Juss. M. Roem.]), also known as *Cedrela sinensis,* Chinese mahogany cedar, or Chinese Toona, is a tree in the Meliaceae family that is widely distributed throughout Asia [1]. The leaves and young shoots of TS can be eaten, and almost every part of TS, including seeds, bark, root bark, petioles, and leaves, have been used medicinally in eastern Asia. The leaves of TS have been found to have anti-inflammatory, analgesic, and antiparasitic effects, and can act as an antidote and inhibit boil growth. In traditional medical systems, TS extracts have been used to treat enteritis, dysentery, carbuncles, boils, dermatitis, scabies, tinea blanca, and itch [2-5]. Aqueous extracts of leaves of TS have also been used to lower blood pressure associated with diabetes. A number of compounds including retinoids, vitamins B and C, *o*-coumaric acid, kaempferol, methyl gallate, quercetin, afzelin, quercitrin, isoquercitrin, and rutin have been isolated from the leaves of TS [5]. Extracts from the leaves of TS exert potent antiproliferative and apoptotic effects on human ovarian and lung cancer cells [6-8]. Methyl gallate isolated from TS was found to have a protective effect in hydrogen peroxide-induced oxidative stress and DNA damage in Madin-Darby canine kidney (MDCK) cells [9]. Moreover, TS can induce lipolysis in differentiated 3 T3-L1 adipocytes and enhance glucose uptake in 3 T3-L1 adipocytes [10,11]. TS was also shown to inhibit viral attachment (influenza A, H1N1) through significant downregulation of adhesion molecules and chemokines (VCAM-1, ICAM-1, E-selectin, IL-8, and fractalkine) and may be used as an alternative treatment and prophylaxis against pandemic influenza A (H1N1) virus [12].

Twenty-seven percent of cancer-related pain originates from the abdominal region [13]. Despite more efficient use of narcotics and intrathecal opioids, visceral pain in patients with abdominal cancer is still difficult to control. A variety of ablative surgeries have been proposed to treat intractable cancer-related abdominal pain, and some neuroablative procedures interrupting the spinothalamic tract and related pathways have been used. However, their use is limited because of significant complications, including a decrease in proprioception, dysesthesia, transient paresis, sphincter dysfunction, and even death [14].

This study was designed to evaluate the antinociceptive effect of different leaf extracts from TS on a visceral pain writhing test in mice.

## Methods

### Plant material

The leaves used in this preparation were obtained from TS grown in Tuku (Yunlin County, Taiwan) and were picked and washed thoroughly with water. A voucher specimen (FY-001) was characterized by Dr. Horng-Liang Lay, Graduate Institute of Biotechnology, National Pingtung University of Science and Technology, Pingtung County, Taiwan, and deposited at Fooyin University, Kaohsiung.

### Preparation and fractionation of TS leaf extracts

Reverse osmosis water was added to the leaves at a proportion of 4 L to 1 kg of leaves. The mixture was heated to a boil and maintained for 30 min. The mixture was then cooled slowly for 2 h at room temperature. The leaves were then removed, and the remaining liquid was concentrated over low heat and filtered with a sieve (70-mesh). The filtered concentrate was lyophilized with a VirTis apparatus (SP Scientific, Gardiner and Stone Ridge, New York, USA) to obtain a crude extract (TSL1). After lyophilization, 100 g of leaves yielded approximately 5–6 g of TSL1 powder. The powder was then dissolved in 99.5% ethanol and centrifuged at 3000 rpm and 4°C (Beckman AvantiTM J-30I) for 12 min to yield a supernatant and pellet. The supernatant was further lyophilized to obtain the powder TSL2. The pellet was further lyophilized and then dissolved in 50% ethanol. The 50% ethanol solution was centrifuged at 4°C and 3000 rpm for 12 min to yield a supernatant and precipitate. The supernatant was lyophilized to obtain the powder TSL3. The precipitate was lyophilized and then dissolved in 25% ethanol. The 25% ethanol solution was centrifuged at 4°C and 3000 rpm for 12 min to yield a supernatant and precipitate. The supernatant was lyophilized to obtain the powder TSL4. Finally, the precipitate was dissolved in reverse osmosis water and centrifuged at 4°C and 3000 rpm for 12 min to yield a supernatant, which was lyophilized to obtain the powder TSL5.

### Animals

A total of 144 male imprinting control region (ICR) mice (body weight = 24–30 g), aged 4–5 weeks, were used in this study. Experimental protocols used in this study were approved by the Kaohsiung Medical University animal research committee. One hundred and twelve animals were fasted overnight before dosing, and were evenly divided into fourteen groups as follows. Group 1, control group (treatment with vehicle, double distilled water 10 ml/kg). Groups 2, 3, 4, 5, and 6 were treated with 1 g/kg TSL1, TSL2, TSL3, TSL4, and TSL5, respectively. Groups 7, 8, 9, and 10 were treated with TSL1 at doses of 0.3, 0.1, 0.03, and 0.01 g/kg, respectively. Groups 11 and 12 were treated with 5- and 1.5-mg/kg doses, respectively, of rofecoxib (a cyclooxygenase-2 [COX-2] inhibitor, Merck Pharmaceuticals Ltd., Whitehouse Station, New Jersey, USA). Groups 13 and 14 were treated with 5- and 10-mg/kg doses, respectively, of diclofenac (Novartis Pharmaceuticals Ltd., Basel, Switzerland). All treatments were administered orally (p.o.), 1 h before intraperitoneal (i.p.) administration of acetic acid 1% (10 mg/kg). Another 32 animals were included in a time-course study. TSL1 (0.1 g/kg) was orally administered 0, 0.5, 2, and 6 h before i.p. administration of 1% acetic acid (10 mg/kg).

### Writhing test

Mice were p.o. administered with their respective treatments. After 1 h, each animal was injected i.p. with 1% acetic acid (10 mg/kg) and individually housed in a glass cylinder on a flat glass floor. Time-course data were obtained by orally administering TSL1 (0.1 g/kg) 0, 0.5, 1, 2, and 6 h before acetic acid administration. Immediately after acetic acid injection, the number of writhes per mouse was counted for 30 min. The writhing activity consists of a contraction of the abdominal muscles together with a stretching of the hind limbs [14]. The percentage of inhibition was calculated using the following ratio: (control mean − treated mean) × 100/control mean.

### Statistical analysis

Results are expressed as mean ± standard deviation (S.D.) and statistically analyzed by analysis of variance, followed by Student’s *t*-test. A probability level lower than 0.05 was considered as statistically significant.

## Results

Antinociceptive effects induced by different doses of TS fractions on the writhing test in mice are shown in Table 1. After treatment with TSL1, TSL2, TSL3, TSL4, and TSL5 at doses of **1 g/kg,** the respective writhing responses were 39.9% (*P <* 0*.*001), 19.5% (*P <* 0*.*05), 11.7% (*P* = 0.052), 8.1% (*P* = 0.188), and 11.4% (*P* = 0.057) lower than that of the control group. Mice treated with TSL1 at 1 g/kg (39.9%, *P <* 0*.*001), 0.3 g/kg (38.0%, *P <* 0*.*001), 0.1 g/kg (46.9%, *P <* 0*.*001), and 0.03 g/kg (31.1%, *P <* 0*.*001) had significantly lower writhing response compared with control mice, with values ranging from 31.1–46.9% inhibition. However, the writhing response in animals treated with 0.01 g/kg TSL1 did not differ significantly when compared with the control (11.7%, *P* = 0.092). The most effective dose of TSL1 (0.1 g/kg p.o.) was used in the time course study, with the effect beginning at 30 min of pre-treatment and persisting until 6 h, as shown in Table 2.

**Table 1:** **Effect of oral administration of TS extract fractions, rofecoxib, and diclofenac on abdominal writhing in mice induced by intraperitoneal injection of acetic acid**

**Dose**	Writhing response*	**Percentage of inhibition**	***P*** **value**
	**(No. of writhes in 30 min)**		
Control by Vehicle (10 ml/kg)	127.8 ± 13.46		
TSL1 (1 g/kg)	76.9 ± 20.9	39.9%	*<* 0*.*001
TSL2 (1 g/kg)	102.9 ± 28.3	19.5%	*<* 0*.*05
TSL3 (1 g/kg)	112.8 ± 14.8	11.7%	0.052
TSL4 (1 g/kg)	117.4 ± 16.4	8.1%	0.188
TSL5 (1 g/kg)	113.3 ± 14.6	11.4%	0.057
TSL1 (0.3 g/kg)	79.3 ± 21.7	38.0%	*<* 0*.*001
TSL1 (0.1 g/kg)	67.8 ± 20.7	46.9%	*<* 0*.*001
TSL1 (0.03 g/kg)	85.0 ± 24.9	31.1%	*<* 0*.*001
TSL1 (0.01 g/kg)	113.1 ± 17.2	11.7%	0.092
Rofecoxib (1.5 mg/kg)	93.4 ± 13.2	26.9%	*<* 0*.*01
Rofecoxib (5 mg/kg)	84.6 ± 11.0	33.8%	*<* 0*.*001
Diclofenac (5 mg/kg)	84.9 ± 13.1	33.5%	*<* 0*.*001
Diclofenac (10 mg/kg)	72.0 ± 14.5	43.7%	*<* 0*.*001

*Values are expressed as mean ± S.D. (*n* = 8).

**Table 2:** **Time-course effect of TSL1 (0.1 g/kg) on the writhing test in mice**

**Pre-treatment (h) with TSL1 (0.1 g/kg)**	No. of writhes in 30 min/writhing response*	**Percentage of inhibition**	***P*** **value**
Control	127.8 ± 13.46	0.0	
0.0	134.8 ± 11.7	−1.1%	0.285
0.5	102.1 ± 22.8	20.1%	< 0.05
1.0	67.8 ± 20.7	46.9%	< 0.001
2.0	85.3 ± 22.6	33.3%	< 0.001
6.0	94.6 ± 14.0	25.9%	< 0.001

*Values are expressed as mean ± S.D. (n = 8).

Treatment with 1.5 and 5 mg/kg rofecoxib reduced writhing responses by 26.9% (*P* < 0.01) and 33.8% (*P* < 0.001), respectively, compared with the control group. Treatment with 5 and 10 mg/kg diclofenac, reduced respective writhing responses by 33.5% (*P* < 0.001) and 43.7% (*P* < 0.001), compared with the control group (Table 1). The antinociceptive effect of TSL1 (0.1 g/kg) is comparable with that of rofecoxib and diclofenac (*P >* 0*.*05).

## Discussion

Cancer-related pain may originate from nociceptors in bone (35%), soft tissue (45%), visceral structures (33%), or may be neuropathic in origin (34%) [13]. Because of the rapid progression of abdominal cancers, it is plausible that most visceral pain is because of visceral organ inflammation. Treatment for visceral pain in advanced cancer patients with either medication or surgery is critical to their quality of life. However, the results of common treatments are unsatisfactory. Clinical use of herbal medicines could be an alternative strategy to treating patients with intractable pain. The present study demonstrated that the fractionated TS leaf extract given orally in mice has analgesic properties in an acetic acid-induced writhing model of nociception. We found that the most significant antinociceptive effect was that of TSL1 at a dose of 0.1 g/kg. Increased TSL1 dosage did not increase the antinociceptive effects. However, TSL1 at a dosage of 0.01 g/kg had an antinociceptive effect that was not significantly different from that of the control group. We hypothesize that TSL1 has various components that interact negatively with increased dosage, which reduces the antinociceptive effect. The results also imply that possible receptor/ligand-gated mechanisms may involve the antinociceptive effect of TSL1. Further study should focus on the component that is responsible for the anti-visceral pain activity of TSL1.

The writhing test is an experimental model used for the screening of drugs with analgesic activity. The model is induced by intraperitoneal injection of acetic acid, which causes irritation. The writhing response is the contraction of the abdominal muscles accompanying an extension of the forelimbs and elongation of the body, which is an established visceral inflammatory pain model [15]. Both central and peripheral analgesic effects can be tested with the writhing test. However, the lack of specificity in the writhing test should be taken into consideration when interpreting these results until other tests have been performed [16,17]. Nevertheless, a good relationship exists between the potencies of analgesics in writhing assays and their clinical potencies. Our present study confirmed the anti-visceral pain properties of TS, which were comparable with those of diclofenac and rofecoxib. Therefore, TS is a candidate for further investigation as an anti-visceral pain drug. However, there is no evidence confirming the interactive effects of TS pain mediators related to visceral pain, including histamine, 5-hydroxytryptamine, kinins, acetylcholine, substance P, and prostaglandins [18,19]. Further study should be conducted to elucidate the exact mechanism of action of TS leaf extract. Although presivous studies with cell-lines showed TS extracts exert potent antiproliferative and apoptotic effects on human ovarian and lung cancer cells [6-8], we did not observe significant side effects or behavioral changes in the present study. Furthermore objective and structured evaluation of behavior and locomotor function also should be included.

The antinociceptive effect of TSL1 was evident 30 min after oral administration and lasted for 6 h. Therefore, our results indicate that the analgesic compounds are absorbed orally, and that pre-systemic metabolism does not impede this effect.

## Conclusions

The results of the present study provide evidence that fractionated TS leaf extracts have antinociceptive effects in a visceral pain mouse model. Further studies should be conducted to elucidate the components responsible for the pharmacological activity.

## Abbreviations

COX-2: cyclooxygenase-2
NSAID: Non-steroidal anti-inflammatory drug
i.p.: Intraperitoneal
p.o.: Per os (oral, by mouth)
TS: *Toona sinensis*
